# miR-128-3p inhibits apoptosis and inflammation in LPS-induced sepsis by targeting TGFBR2

**DOI:** 10.1515/med-2021-0222

**Published:** 2021-02-04

**Authors:** Peng Yang, Jianhua Han, Shigeng Li, Shaoning Luo, Xusheng Tu, Zhiqiang Ye

**Affiliations:** Department of Anesthesiology, The First Affiliated Hospital, Sun Yat-sen University, 510080, Guangzhou, China; Department of Emergency, The Third Affiliated Hospital, Sun Yat-sen University, No. 600 Tianhe Road, Tianhe District, 510630, Guangzhou, China

**Keywords:** sepsis, miR-128-3p, TGFBR2, apoptosis, inflammation

## Abstract

**Background:**

Sepsis is a systemic inflammatory response that can lead to the dysfunction of many organs. The aberrant expression of miRNAs is associated with the pathogenesis of sepsis. However, the biological functions of miR-128-3p in sepsis remain largely unknown, and its mechanism should be further investigated. This study aimed to determine the regulatory network of miR-128-3p and TGFBR2 in lipopolysaccharide (LPS)-induced sepsis.

**Methods:**

The expression levels of miR-128-3p and transforming growth factor beta receptors II (TGFBR2) were detected by quantitative polymerase chain reaction (qPCR). The protein levels of TGFBR2, Bcl-2, Bax, cleaved caspase 3, Smad2, and Smad3 were measured by western blot. Cell apoptosis was analyzed by flow cytometry. Cytokine production was detected by enzyme-linked immunosorbent assay (ELISA). The binding sites of miR-128-3p and TGFBR2 were predicted by Targetscan online software and confirmed by dual-luciferase reporter assay and RNA immunoprecipitation (RIP) assay.

**Results:**

The level of miR-128-3p was decreased, and TGFBR2 expression was increased in serum samples of sepsis patients and LPS-induced HK2 cells. Overexpression of miR-128-3p or knockdown of TGFBR2 ameliorated LPS-induced inflammation and apoptosis. Moreover, TGFBR2 was a direct target of miR-128-3p, and its overexpression reversed the inhibitory effects of miR-128-3p overexpression on inflammation and apoptosis in LPS-induced HK2 cells. Besides, overexpression of miR-128-3p downregulated TGFBR2 to suppress the activation of the Smad signaling pathway.

**Conclusion:**

miR-128-3p could inhibit apoptosis and inflammation by targeting TGFBR2 in LPS-induced HK2 cells, which might provide therapeutic strategy for the treatment of sepsis.

## 1 Introduction

Sepsis is a life-threatening organ dysfunction caused by a dysregulated host response to infection [1,2]. Although there have been significant improvements in critical care medicine in recent years, sepsis remains one of the leading causes of mortality in the intensive care unit (ICU) [3,4]. Therefore, it is critical to explain the pathogenic mechanisms at the molecular level, providing a potentially effective treatment for sepsis.

MicroRNAs (miRNAs) are a kind of small noncoding RNAs that regulate the expression of protein through targeting the 3′UTR of messenger RNA (mRNA), resulting in mRNA degradation or suppression of translation [5]. Increasing evidence has suggested that the dysregulation of miRNAs is associated with the pathogenesis of sepsis. For example, serum miR-146a and miR-223 were evidently decreased in septic patients compared with healthy controls [6]. In addition, miR-27a expression was increased and promoted inflammatory response in sepsis [7]. miR-128-3p was recently reported to be downregulated in several types of cancer, such as hepatocellular carcinoma and lung cancer [8,9]. Besides, it has been reported that the expression of miR-128 was decreased in podocytes of a sepsis patient [10]. However, the biological function of miR-128-3p in sepsis has not been well elucidated.

Identification of miRNA-regulated targeting genes is important for understanding their specific biological functions [11]. Transforming growth factor-β (TGF-β) signaling pathway is a multifunctional cytokine and plays important roles in regulating cell proliferation, cell cycle, differentiation, migration, and apoptosis in a broad spectrum of tissues [12]. TGF-β signaling is initiated when the ligand binds to type II serine/threonine kinase receptor (TGFBR2), which then phosphorylates and activates type I serine/threonine kinase receptor (TGFBR1) [13]. The activated type I receptor can activate Smad proteins that regulate transcription [14]. A previous study has shown that the blood level of TGF-β could distinguish between H1N1 virus sepsis and sepsis due to other forms of community-acquired pneumonia [15]. In addition, levosimendan upregulated TGF-β and Smad signaling in the aorta in the early stage of sepsis [16]. These studies revealed that TGFBR2 might play important roles in sepsis. Therefore, the underlying mechanisms of TGFBR2 in sepsis should be clearly elucidated. Interestingly, online bioinformatics database showed that TGFBR2 had complementary binding sites for miR-128-3p, which prompted us to construct a miRNA-mRNA regulatory network in sepsis.

In this study, the levels of miR-128-3p and TGFBR2 in serum samples of sepsis patients and LPS-induced HK2 cells were detected. Moreover, we investigated the effects of miR-128-3p on apoptosis and inflammation and explored the regulatory network of miR-128-3p and TGFBR2 in lipopolysaccharide (LPS)-induced HK2 cells. In conclusion, this study might provide potential therapeutic value for sepsis treatment.

## 2 Materials and methods

### 2.1 Patients and blood samples collection

All blood samples were obtained from The First Affiliated Hospital of Sun Yat-sen University. All participants did not receive chemotherapy or radiotherapy. Human blood samples were obtained from 15 patients with sepsis (mean age: 56.27 ± 7.11, 66.67% male) following the definitions of the American College of Chest Physicians/Society of Critical Care Conference [17]. Fifteen age- and sex-matched healthy volunteers (mean age: 56.27 ± 7.11, 66.67% male) served as control subjects. This study was granted by the ethics committee of The First Affiliated Hospital of Sun Yat-sen University, and written informed consent was obtained from all participants.

### 2.2 Serum total RNA isolation

In brief, special tubes containing separating gel and clot activator were used to collect blood samples and then centrifuged at 3,000 rpm for 15 min at room temperature. The supernatant was transferred to clean Eppendorf tubes and centrifuged at 1,500 rpm for 30 min to remove cell debris. The final supernatant was stored at −80°C until RNA extraction. Serum total RNA including miRNAs was isolated using mirVana miRNA Isolation Kit (Ambion, Austin, TX, USA), and the serum total mRNA was extracted by using the RNeasy Mini Kit (Qiagen, Valencia, CA, USA). All procedures followed the manufacturer’s protocol for liquid samples.

### 2.3 Cell culture and transfection

Human kidney-2 (HK2) cell line was purchased from American Tissue Culture Collection (ATCC; Manassas, VA, USA). HK2 cells were cultured in Dulbecco’s modified eagle medium (DMEM; Hyclone, Logan, UT, USA) supplemented with 10% fetal bovine serum (FBS; Gibco, Carlsbad, CA, USA) in an incubator with 5% CO_2_ at 37°C. We used HK2 cells induced by LPS (10 μg/mL) to establish the sepsis model.

miR-128-3p mimic (miR-128-3p) and mimic negative control (NC; a random sequence miRNA mimic molecule), miR-128-3p inhibitor (anti-miR-128-3p) and inhibitor negative control (anti-NC; a chemically modified single-stranded nucleic acid of random sequence), small interfering RNA (siRNA) against TGFBR2 (si-TGFBR2) and siRNA negative control (Scramble, nonspecific scramble siRNA), TGFBR2 overexpression vector (TGFBR2), and vector negative control (vector, transfected empty vector) were obtained from GenePharma (Shanghai, China) and transfected into HK2 cells using Lipofectamine 3000 (Invitrogen, Carlsbad, CA, USA) according to the manufacturer’s protocol.

### 2.4 Quantitative polymerase chain reaction

Total RNA was isolated from cells using Trizol reagent (Invitrogen) following the manufacturer’s instructions. Complementary DNA (cDNA) was synthesized from total RNA by using TaqMan Reverse Transcription Kit or TaqMan microRNA Reverse Transcription Kit (Applied Biosystems, Foster City, CA, USA). Subsequently, qPCR was conducted using SYBR Green PCR Kit (Toyobo, Tokyo, Japan) on ABI Prism 7500 Detection System (Applied Biosystems). U6 or GAPDH served as an internal reference for miR-128-3p or TGFBR2, respectively. The relative expression of mRNAs was evaluated with the 2^−ΔΔCt^ method. The primers were obtained from Sangon Biotech (Shanghai, China), and primer sequences were listed below: miR-128-3p (forward, 5′-GACTGCCGAGCGAGCG-3′; reverse, 5′-GACGCCGAGGCACTCTCTCCT-3′), U6 (forward, 5′-CCATCGGAAGCTCGTATACGAAATT-3′; reverse, 5′-GGCCTCTCGAACTTGCGTGTCAG-3′), TGFBR2 (forward, 5′-GCTGATCACCGCCTTCCA-3′; reverse, 5′-CAGGTCCTCCCAGCTGATGA-3′), and GAPDH (forward, 5′-CCACATCGCTCAGACACCAT-3′; reverse, 5′-GCG CCCAATACGACCAAAT-3′).

### 2.5 Western blot assay

Total protein was extracted from collected cells using radio-immunoprecipitation assay (RIPA) lysis buffer (Thermo Fisher Scientific, Wilmington, DE, USA) containing the protease inhibitors (Roche, Basel, Switzerland) and then centrifuged at 12,000 rpm for 15 min to collect the supernatant. Next, protein samples were quantified using bicinchoninic acid (BCA) protein assay kit (Beyotime Biotechnology, Shanghai, China) and boiled for 10 min with 2× loading buffer (Beyotime Biotechnology). Subsequently, an equivalent protein in each sample was loaded and separated on 10–12% sodium dodecyl sulfate-polyacrylamide gel electrophoresis (SDS-PAGE) and then transferred to polyvinylidene difluoride (PVDF) membranes (Millipore, Billerica, MA, USA). The membranes were then blocked with 5% nonfat milk in Tris-buffer saline containing 0.1% Tween 20 (TBST; 1 h, room temperature) and then probed with a specific primary antibody (4°C, overnight) against TGFBR2 (1:1,000, ab186838, Abcam, Cambridge, UK), Bcl-2 (1:1,000, ab196495, Abcam), Bax (1:1,000, ab199677, Abcam), cleaved caspase 3 (1:500, ab49822, Abcam), Smad2 (1:2,000, ab40855, Abcam), Smad3 (1:2,000, ab40854, Abcam), or GAPDH (1:2,500, ab9485, Abcam). Then, the membranes were washed with TBST three times and incubated with horseradish peroxidase (HRP)-conjugated secondary antibodies (1:4,000, Sangon Biotech, Shanghai, China) for 2 h at room temperature. Finally, the protein bands were visualized by the enhanced chemiluminescence (ECL) system (Thermo Fisher Scientific) in the dark and quantitated using ImageJ software (National Institutes of Health, Bethesda, MD, USA).

### 2.6 Cell apoptosis assay

Cell apoptosis was measured by flow cytometry with Annexin V-fluorescein isothiocyanate (FITC)/propidium iodide (PI) apoptosis detection kit (Sigma, St. Louis, MO, USA). In brief, treated or transfected HK2 cells were seeded into six-well plates. After 48 h, cells were collected and stained with Annexin V-FITC and PI for 20 min in a dark place. Finally, cell apoptosis was detected using the flow cytometry (B.D. FACS Calibur) and analyzed by the flow cytometer software.

### 2.7 Enzyme-linked immunosorbent assay

The concentrations of TNF-α and IL-2 were measured by using commercially available enzyme-linked immunosorbent assay (ELISA) kits (R & D Systems Inc., Minneapolis, MN, USA) following the manufacturer’s instructions.

### 2.8 Dual-luciferase reporter assay

The putative binding sites of miR-128-3p and TGFBR2 were predicted by online software Targetscan (http://www.targetscan.org/cgi-bin/targetscan/vert_71/view_gene.cgi?rs=ENST00000359013.4&taxid=9606&members=miR-128-3p&showcnc=0&shownc=0&subset=1). The sequences of wild-type TGFBR2 (TGFBR2-wt) and mutant TGFBR2 (TGFBR2-mut) with predicted binding sites to human miR-128-3p were amplified and cloned into the pGL3 luciferase reporter vectors (Promega, Madison, WI, USA). The constructed pGL3 vectors were then co-transfected with NC or miR-128-3p into HK2 cells according to the manufacturer’s protocols. After transfection for 48 h, the luciferase activities were analyzed by Dual-Luciferase Assay Kit (Promega) and normalized to the Renilla luciferase activity.

### 2.9 RNA Immunoprecipitation assay

The relationship between miR-128-3p and TGFBR2 was measured by Magna RIP Kit (Millipore) following the manufacturer’s instructions. In brief, HK2 cells transfected with miR-128-3p or NC were collected and resuspended in RNA immunoprecipitation lysis buffer containing magnetic beads and then incubated with anti-argonaute 2 (anti-Ago2) or IgG antibodies. Subsequently, the protein was digested through proteinase K buffer, followed by RNA purification. Finally, the purified RNA was used for the qPCR analysis of the TGFBR2 level.

### 2.10 Statistical analysis

The data were presented as the mean ± standard deviation (SD) from at least three independent experiments. Spearman rank correlation was performed to explore the correlation between miR-128-3p and TGFBR2. Statistical analyses were performed using Graphpad Prism version 6.0 software (GraphPad Software, San Diego, CA, USA). The differences between two groups were assessed using the two-tailed Student’s *t* test. *P* < 0.05 was considered to indicate a statistically significant difference.

## 3 Results

### 3.1 The expression of miR-128-3p was decreased and TGFBR2 expression was increased in serum samples with sepsis and LPS-induced HK2 cells

**Figure 1:**
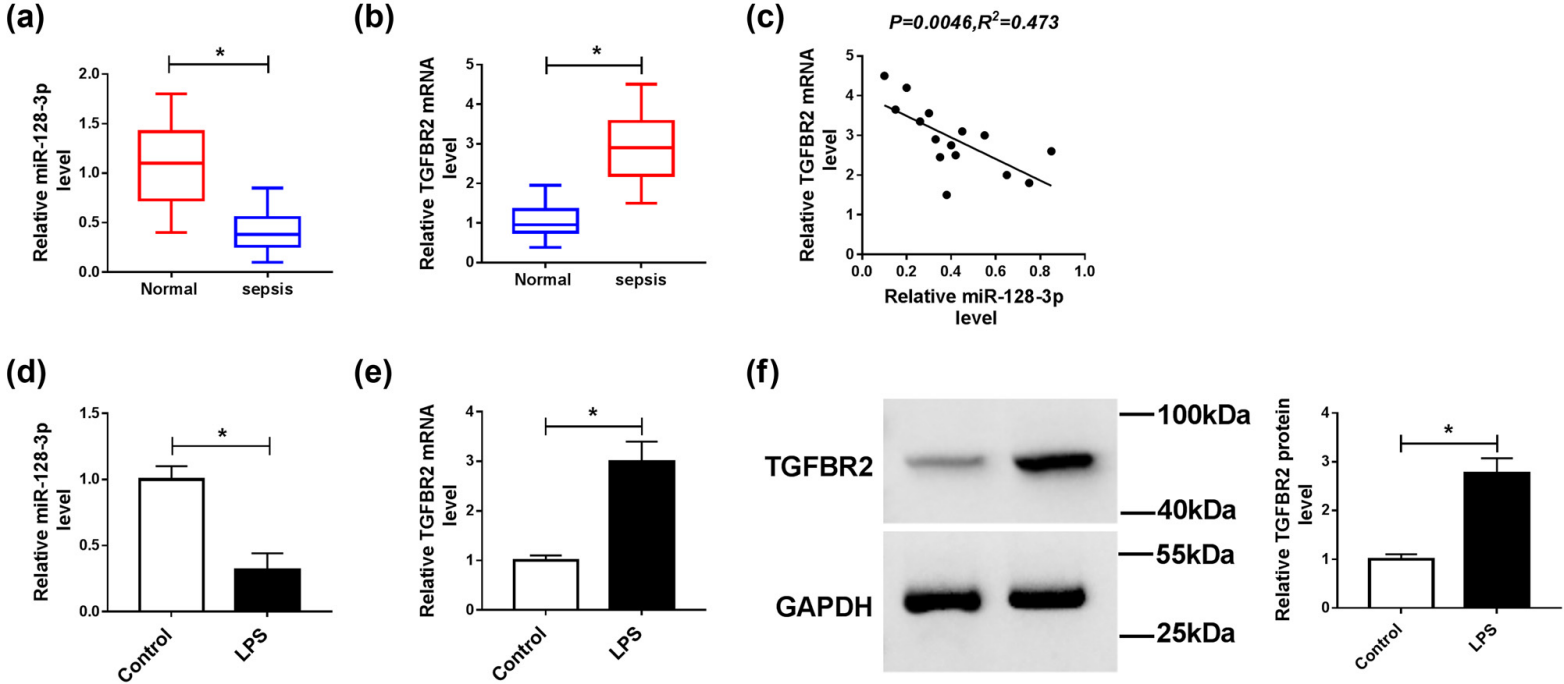
Expression levels of miR-128-3p and TGFBR2 in serum samples of sepsis patients and LPS-induced HK2 cells. (a and b) The expression levels of miR-128-3p and TGFBR2 were measured in serum samples of patients with sepsis and control groups by qPCR. (c) The association between TGFBR2 mRNA level and miR-128-3p abundance was measured in serum samples of patients with sepsis. (d and e) The expression levels of miR-128-3p and TGFBR2 were detected in LPS-induced HK2 cells by qPCR. (f) Western blot was performed to detect the protein level of TGFBR2. *P < 0.05.

To explore the roles of miR-128-3p and TGFBR2 in sepsis, we determined their levels by qPCR in serum samples from patients with sepsis. Results proved that the level of miR-128-3p was markedly downregulated, and the expression of TGFBR2 was notably upregulated in serum samples of patients with sepsis compared with control groups (Figure 1a and b). In addition, the relationship between miR-128-3p and TGFBR2 was analyzed in serum samples of patients with sepsis. We found that the level of TGFBR2 was negatively associated with miR-128-3p expression (Figure 1c). Next, we detected the levels of miR-128-3p and TGFBR2 in the LPS-induced HK2 cells. The results suggested that the abundance of miR-128-3p was remarkably decreased in the LPS-induced HK2 cells compared with control cells (Figure 1d). Besides, the mRNA and protein levels of TGFBR2 were apparently elevated in the LPS-treated HK2 cells (Figure 1e and f). These findings indicated that miR-128-3p and TGFBR2 might play important roles in the development of sepsis.

### 3.2 Overexpression of miR-128-3p reversed the effects of LPS on apoptosis and inflammation in HK2 cells

**Figure 2:**
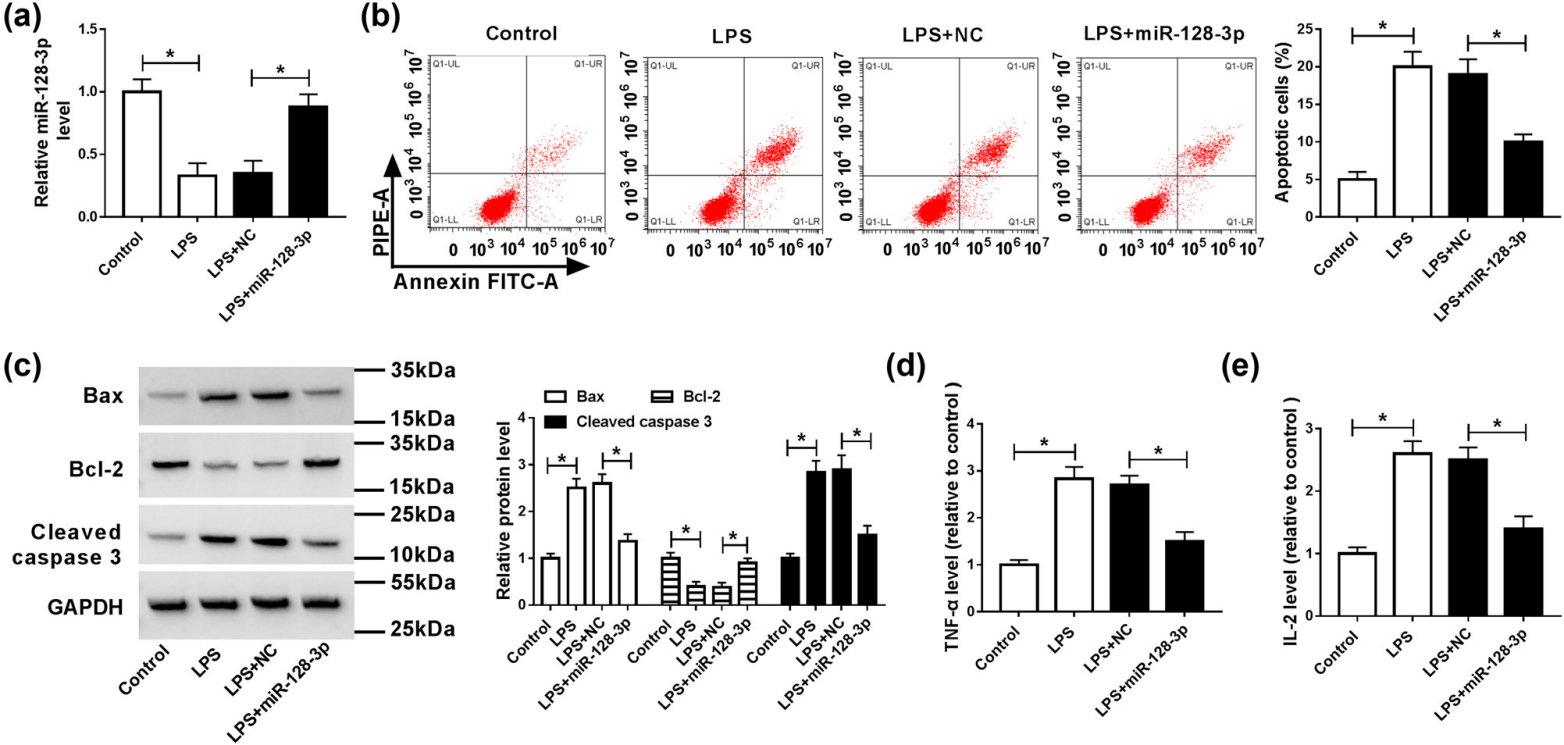
Effects of miR-128-3p on apoptosis and inflammation in LPS-induced HK2 cells. HK2 cells were treated with LPS or/and transfected with miR-128-3p. (a) The expression level of miR-128-3p was measured by qPCR. (b) Cell apoptosis was detected by flow cytometry. (c) The protein levels of Bax, Bcl-2, and cleaved caspase 3 were analyzed by western blot. (d and e) The production pro-inflammatory cytokines (TNF-α and IL-2) was detected by ELISA kit. *P < 0.05.

To analyze the effects of miR-128-3p on the LPS-induced apoptosis and inflammatory response in HK2 cells, miR-128-3p was transfected into the LPS-induced HK2 cells. The results of qPCR analysis showed that LPS exposure significantly decreased the level of miR-128-3p, which was abolished by the addition of miR-128-3p (Figure 2a). Moreover, the apoptotic rate was remarkably increased in HK2 cells treated with LPS compared with the control cells, whereas it was ablated by upregulation of miR-128-3p (Figure 2b). Besides, apoptosis-related proteins were analyzed by western blot. The results suggested that LPS treatment prominently increased the protein levels of Bax and cleaved caspase 3 but reduced the protein expression of Bcl-2, which was reversed by accumulation of miR-128-3p (Figure 2c). Furthermore, levels of pro-inflammatory cytokines (TNF-α and IL-2) were evidently elevated in HK2 cells induced with LPS, while overexpression of miR-128-3p overturned this effect (Figure 2d and e). Altogether, these data indicated that miR-128-3p reversed LPS-induced apoptosis and inflammatory response in HK2 cells.

### 3.3 Knockdown of TGFBR2 had the similar effects with overexpression of miR-128-3p in LPS-induced HK2 cells

**Figure 3:**
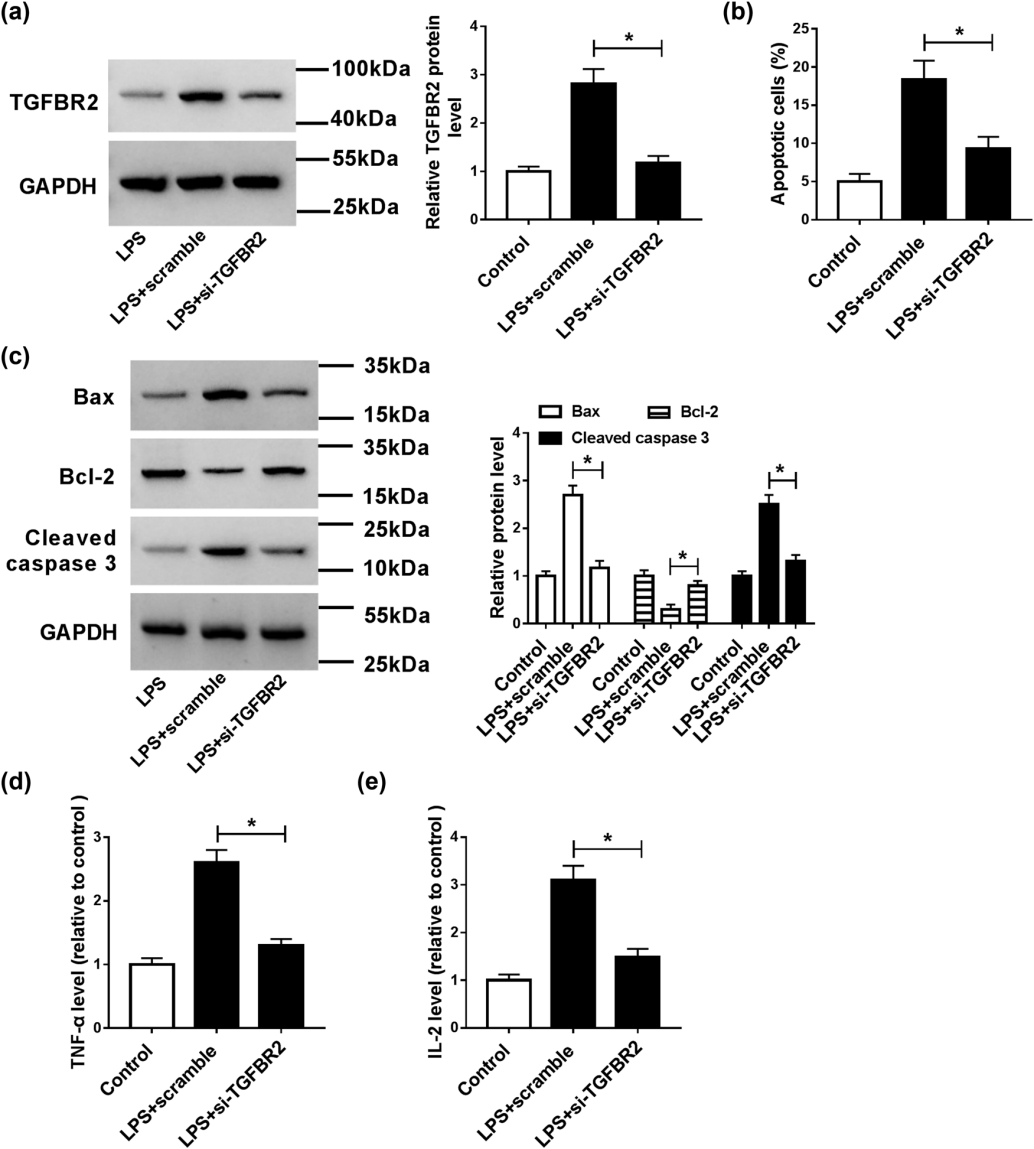
Effects of TGFBR2 on apoptosis and inflammation in LPS-induced HK2 cells. HK2 cells were treated with LPS or/and transfected with si-TGFBR2. (a) The protein level of TGFBR2 was measured by western blot analysis. (b) Cell apoptosis was detected by the flow cytometry. (c) The protein levels of Bax, Bcl-2, and cleaved caspase 3 were analyzed by western blot. (d and e) ELISA kit was used to determine the production of pro-inflammatory cytokines (TNF-α and IL-2). *P < 0.05.

To further study the function of TGFBR2 in LPS-induced HK2 cells, si-TGFBR2 was transfected into HK2 cells treated with LPS. Results revealed that TGFBR2 knockdown reversed LPS-mediated promotion of TGFBR2 expression in HK2 cells (Figure 3a). Moreover, abrogation of TGFBR2 abolished LPS-induced apoptosis in HK2 cells (Figure 3b). Besides, downregulation of TGFBR2 alleviated LPS-mediated promotion of Bax and cleaved caspase 3 protein levels and reduction of Bcl-2 expression (Figure 3c). Furthermore, inhibition of TGFBR2 also weakened the promotion of inflammatory cytokines TNF-α and IL-2 levels caused by LPS (Figure 3d and e). Thus, these data proved that inhibition of TGFBR2 attenuated LPS-induced apoptosis and inflammatory response in HK2 cells.

### 3.4 TGFBR2 was a direct target of miR-128-3p

**Figure 4:**
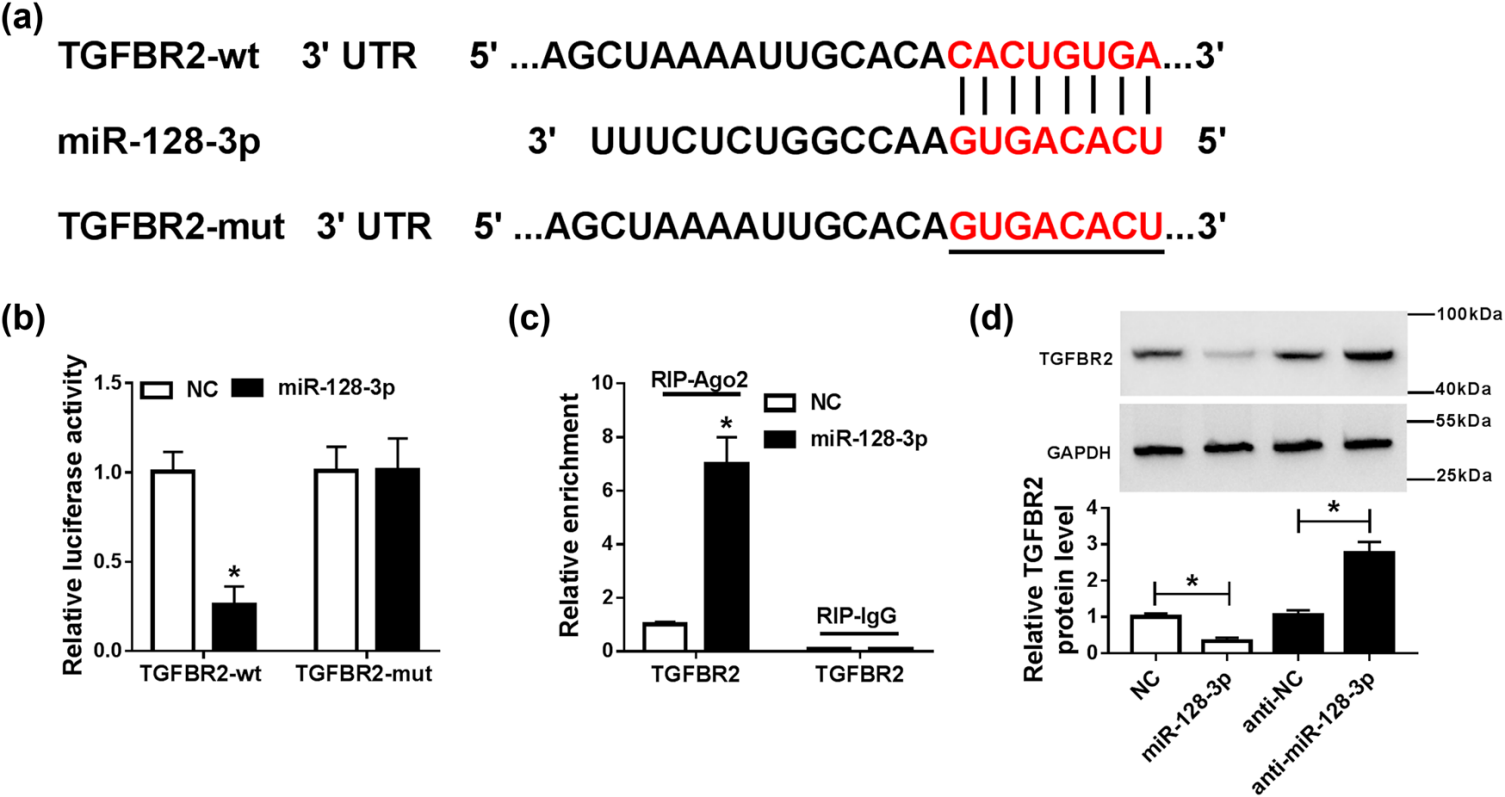
miR-128-3p directly targeted TGFBR2 in HK2 cells. (a) The putative binding sites of miR-128-3p and TGFBR2 were provided by TargetScan. (b) Luciferase activity was measured in HK2 cells co-transfected with TGFBR2-wt or TGFBR2-mut and miR-128-3p or NC by dual-luciferase reporter assay. (c) RIP assay was performed to detect TGFBR2 enrichment level in HK2 cells transfected with miR-128-3p or NC. *P < 0.05. (d) The protein level of TGFBR2 was measured in HK2 cells transfected with miR-128-3p, NC, anti-miR-128-3p, or anti-NC by western blot analysis. *P < 0.05.

To further explore the relationship between miR-128-3p and TGFBR2 in sepsis, the potential binding sites of miR-128-3p and TGFBR2 were predicted by Targetscan online website, indicating that TGFBR2 might be a target of miR-128-3p (Figure 4a). Subsequently, the prediction was confirmed by dual-luciferase reporter assay and RNA immunoprecipitation (RIP) analysis in HK2 cells. Results suggested that transfection of miR-128-3p obviously decreased the luciferase activity of TGFBR2-wt, whereas luciferase activity of TGFBR2-mut was not evidently affected after transfection with miR-128-3p (Figure 4b). RIP analysis suggested that TGFBR2 was notably enriched in miR-128-3p group coated with Ago2 antibody compared with the control group (Figure 4c). Besides, upregulation of miR-128-3p apparently reduced the protein expression of TGFBR2 in HK2 cells, and its knockdown presented an opposite effect (Figure 4d). Taken together, these data indicated that miR-128-3p directly targeted TGFBR2 in HK2 cells.

### 3.5 Upregulation of TGFBR2 reversed the effects of miR-128-3p overexpression in LPS-induced HK2 cells

**Figure 5:**
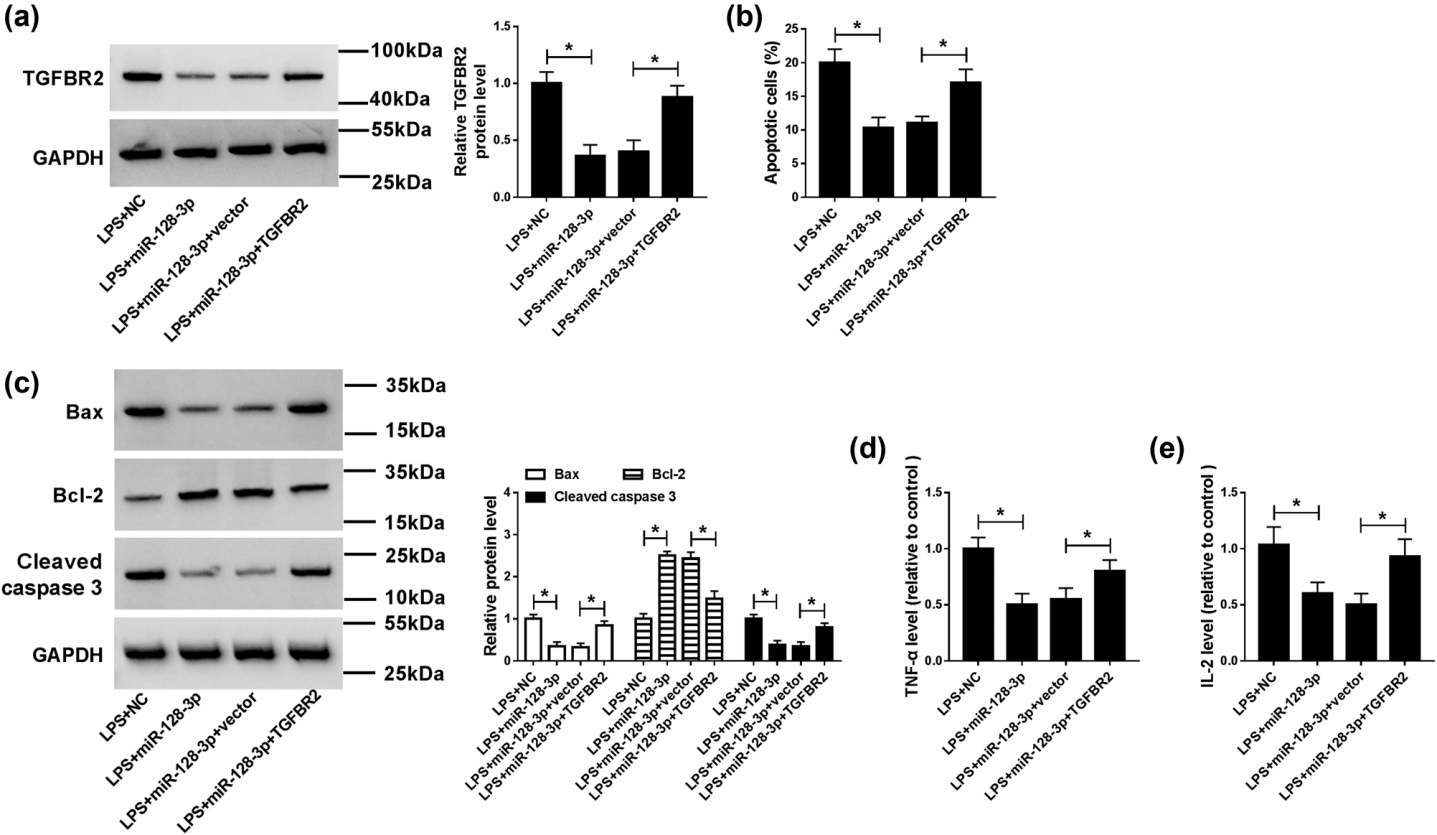
Upregulation of TGFBR2 weakened the effects of miR-128-3p overexpression on apoptosis and inflammation in LPS-induced HK2 cells. miR-128-3p, NC, miR-128-3p + vector, or miR-128-3p + TGFBR2 was transfected into LPS-induced HK2. (a) The protein level of TGFBR2 was detected by western blot analysis. (b) Cell apoptosis was measured by flow cytometry. (c) The protein levels of Bax, Bcl-2, and cleaved caspase 3 were analyzed by western blot. (d and e) The production of pro-inflammatory cytokines (TNF-α and IL-2) was detected by ELISA kit. *P < 0.05.

To explore whether TGFBR2 was involved in miR-128-3p overexpression-mediated inhibition of progression of sepsis, NC, miR-128-3p, miR-128-3p + vector, or miR-128-3p + TGFBR2 was transfected into the LPS-induced HK2 cells. As shown in Figure 5a, overexpression of miR-128-3p prominently reduced the protein level of TGFBR2 in the LPS-induced HK2 cells, which could be abated by the addition of TGFBR2. Moreover, upregulation of TRAF3 abolished the inhibitory effect of miR-128-3p overexpression on apoptosis in LPS-induced HK2 cells (Figure 5b). Besides, transfection of TGFBR2 reversed the effects of miR-128-3p upregulation on reduction of Bax and cleaved caspase 3 expression and promotion of Bcl-2 expression in LPS-induced HK2 cells (Figure 5c). In addition, upregulation of TGFBR2 attenuated the effect of miR-128-3p overexpression on the decreased levels of inflammatory cytokine TNF-α and IL-2 (Figure 5d and e). Altogether, these data indicated that upregulation of TGFBR2 reversed the effects of miR-128-3p overexpression on apoptosis and inflammatory response in the LPS-induced HK2 cells.

### 3.6 miR-128-3p suppressed the activation of the Smad signaling pathway by affecting TGFBR2 expression

**Figure 6:**
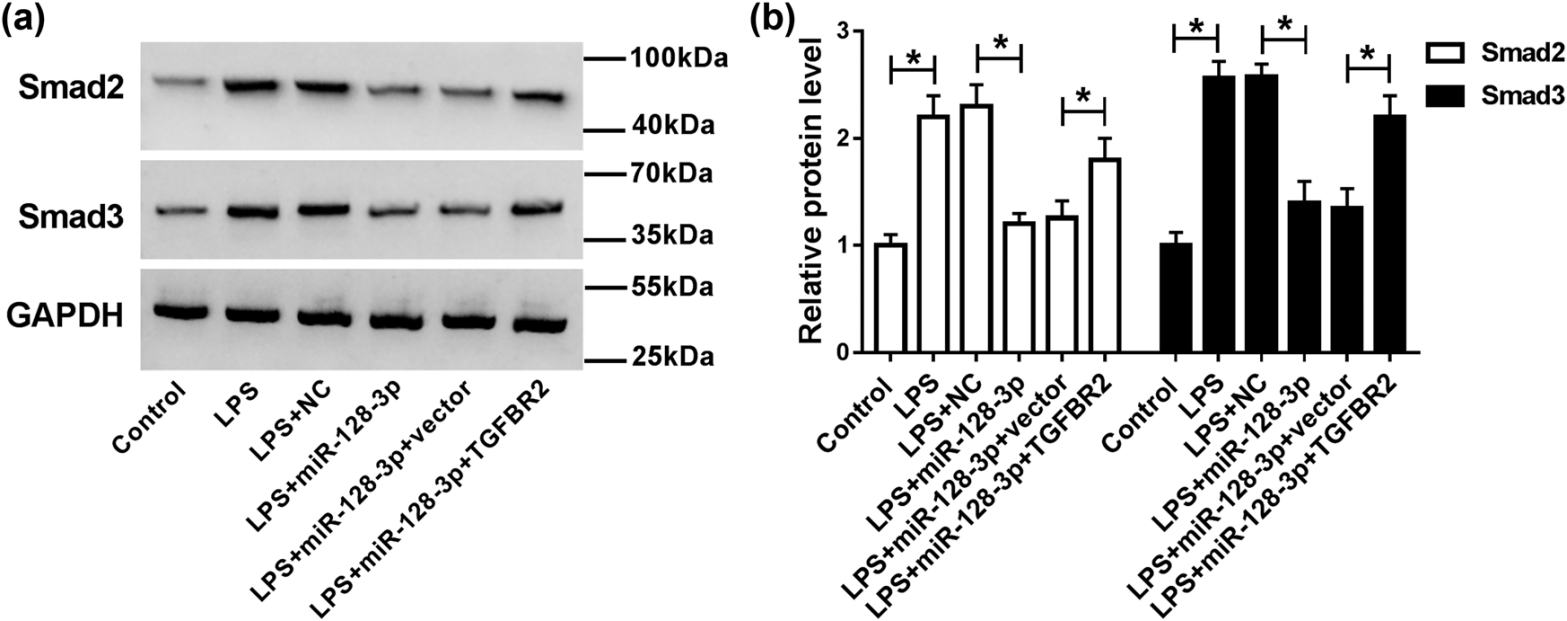
miR-128-3p regulated Smad singling pathway by affecting TGFBR2 expression. LPS-induced HK2 cells were transfected with miR-128-3p, NC, miR128-3p + TGFBR2, or miR128-3p + vector. (a and b) The protein levels of Smad2 and Smad3 were detected by western blot analysis. *P < 0.05.

Smad signaling pathway may play an essential role in the development and progression of sepsis. To further explore the molecular mechanism by which miR-128-3p regulates the biological functions in sepsis, the expression levels of proteins related to the Smad signaling pathway (Smad2 and Smad3) were measured in controls cells and LPS-induced HK2 cells transfected with NC, miR-128-3p, miR-128-3p + vector, or miR128-3p + TGFBR2. Western blot indicated that overexpression of miR-128-3p reversed the LPS-induced promotion of Smad2 and Smad3 protein levels of in HK2 cells, whereas addition of TGFBR2 abolished the effects of miR-128-3p overexpression on expression of Smad2 and Smad3 (Figure 6a and b). From aforementioned outcomes, we demonstrated that miR-128-3p could regulate Smad singling pathway via affecting TGFBR2 expression.

## 4 Discussion

Sepsis is a systemic inflammation response syndrome, which frequently causes extensive tissue injury and multiple organ dysfunctions [18]. If sepsis is not controlled timely and effectively, it can lead to multiple organ dysfunction syndrome, which is the main cause of death in trauma, burn, and critical surgical patients. Recent reports have demonstrated that aberrant expression of miRNAs might be used to improve diagnosis and treatment of sepsis. Hence, it is particularly important to understand the underlying molecular mechanisms of miRNAs for the treatment of sepsis.

miR-128-3p has been suggested to be dysregulated in many conditions and participate in multiple cell behaviors, including proliferation, migration and invasion, epithelial-mesenchymal transition (EMT), and angiogenesis [8,9,19,20]. Increasing evidence suggested that miR128-3p has also been regarded as a prognostic marker in many diseases [20,21,22]. In addition, miR-128-3p could regulate inflammatory response in LPS-stimulated macrophages via the TLR4-NF-κB pathway [23]. Besides, miR-128 specifically inhibited the development of inflammation by downregulation of MyD88 [24]. Previous study had revealed that decreased level of miR-128 synergistically promoted cell injuries through targeting Snail and PTEN [10]. As mentioned earlier, miR-128 could regulate inflammatory responses and might play important roles in sepsis. Consistent with the previous results, the expression of miR-128-3p was decreased in serum samples of sepsis patients and LPS-induced HK2 cells. Moreover, treatment of LPS promoted apoptosis and inflammation in HK2 cells, which was abated by overexpression of miR-128-3p. These findings suggested that miR-128-3p might play vital roles in sepsis.

Increasing evidence shows that miRNAs exert their functions through directly binding to target mRNAs and inhibiting mRNA stability and translation [25]. In our study, we found that TGFBR2 was a direct target of miR-128-3p, and the level of TGFBR2 was negatively associated with miR-128-3p expression. TGFBR2 has been reported to play essential roles in sepsis. Ma et al. demonstrated that TGFBR2/Smad2/DNMT1/miR-145 negative regulatory loop might be a potential target for the treatment of sepsis [26]. Zhang et al. showed that SUMO protease SENP1 acted as a ceRNA for TGFBR2, which activated TGFBR2/Smad signaling responsible for LPS-induced sepsis [27]. Cao et al. identified that the expression TGFBR2 was obviously increased with LPS treatment in a time-dependent manner, and miR-145 ameliorated sepsis-induced lung injury by inhibiting TGFBR2 signaling [28]. Here, we found that knockdown of TGFBR2 ameliorated LPS-induced inflammation and apoptosis. Besides, upregulation of TGFBR2 abrogated miR-128-3p overexpression-mediated attenuation on LPS-induced sepsis, suggesting that miR-128-3p was involved in the development and progression of sepsis by regulation of TGFBR2.

TGF-β1 exerts its biological functions via activating Smad2 and Smad3 [29]. Recently, it had been reported that inhibition of the TGF-β1/Smad3 pathway might play a protective role in sepsis-induced acute lung injury [30]. Besides, propofol could provide protection against acute lung injury through suppressing the TGF-β1-Smad2-dependent pathway [28]. In this study, we found that transfection of miR-128-3p reversed the LPS-induced promotion of Smad2 and Smad3 protein levels of in HK2 cells, whereas addition of TGFBR2 abolished the effects of overexpression of miR-128-3p on expression of Smad2 and Smad3. These results indicated that miR-128-3p could regulate Smad singling pathway by affecting TGFBR2 expression.

In conclusion, we demonstrated that miR-128-3p was decreased and TGFBR2 expression was increased in serum samples of sepsis patients and LPS-induced HK2 cells. Overexpression of miR-128-3p or knockdown of TGFBR2 ameliorated LPS-induced inflammation and apoptosis. Moreover, our study presented the first evidence that TGFBR2 was a direct target of miR-128-3p, and its overexpression reversed the effects of miR-128-3p overexpression on inflammation and apoptosis. Besides, overexpression of miR-128-3p downregulated TGFBR2 to suppress the activation of the Smad signaling pathway. Collectively, miR-128-3p could inhibit apoptosis and inflammation by targeting TGFBR2 in LPS-induced HK2 cells, providing viable therapeutic avenues for the treatment of sepsis.
